# A Sensorised Surgical Glove to Analyze Forces During Neurosurgery

**DOI:** 10.1227/neu.0000000000002239

**Published:** 2022-12-12

**Authors:** Hugo Layard Horsfall, Carmen Salvadores Fernandez, Biswajoy Bagchi, Priyankan Datta, Priya Gupta, Chan Hee Koh, Danyal Khan, William Muirhead, Adrien Desjardins, Manish K. Tiwari, Hani J. Marcus

**Affiliations:** ‡ Victor Horsley Department of Neurosurgery, National Hospital for Neurology and Neurosurgery, London, UK;; § Wellcome/EPSRC Centre for Interventional and Surgical Sciences, University College London, London, UK;; ‖ Nanoengineered Systems Laboratory, UCL Mechanical Engineering, London, UK

**Keywords:** Neurosurgery, Innovation, Force, Intraoperative forces, Education, Instrument, Nanoengineering

## Abstract

**BACKGROUND:**

Measuring intraoperative forces in real time can provide feedback mechanisms to improve patient safety and surgical training. Previous force monitoring has been achieved through the development of specialized and adapted instruments or use designs that are incompatible with neurosurgical workflow.

**OBJECTIVE:**

To design a universal sensorised surgical glove to detect intraoperative forces, applicable to any surgical procedure, and any surgical instrument in either hand.

**METHODS:**

We created a sensorised surgical glove that was calibrated across 0 to 10 N. A laboratory experiment demonstrated that the sensorised glove was able to determine instrument-tissue forces. Six expert and 6 novice neurosurgeons completed a validated grape dissection task 20 times consecutively wearing the sensorised glove. The primary outcome was median and maximum force (N).

**RESULTS:**

The sensorised glove was able to determine instrument-tissue forces reliably. The average force applied by experts (2.14 N) was significantly lower than the average force exerted by novices (7.15 N) (*P* = .002). The maximum force applied by experts (6.32 N) was also significantly lower than the maximum force exerted by novices (9.80 N) (*P* = .004). The sensorised surgical glove's introduction to operative workflow was feasible and did not impede on task performance.

**CONCLUSION:**

We demonstrate a novel and scalable technique to detect forces during neurosurgery. Force analysis can provide real-time data to optimize intraoperative tissue forces, reduce the risk of tissue injury, and provide objective metrics for training and assessment.

**ABBREVIATIONS:**
CNF: carbon nanofiber
N: Newton.

Surgery structurally alters the human body by the incision or destruction of tissues.^1^ Surgery involves instrument-tissue interactions that enable specific manipulation of target tissue through physical forces applied by instruments, such as a scalpel or forceps. Uncontrolled or excessive instrument-tissue interaction forces can lead to tissue damage and intraoperative complications, while insufficient forces prevent task completion.^2^ The knowledge of instrument-tissue interaction is mostly learnt through the novice/expert model,^3^ and neurosurgeons spend years undertaking deliberate practice to master psychomotor skills.^4(p)^ A challenging aspect of training is to acquire knowledge of the optimal instrument force necessary to complete a given surgical task.^3^ Force analysis of instrument-tissue interaction may also help distinguish the surgeon's skill level, which could enhance surgical education as it shifts to a competency-based paradigm.^4^ Measuring forces during surgery may provide objective metrics for training and assessment.^2^ It may also contribute to patient safety, as even expert surgeons under duress can exert increased forces if under duress or fatigued.^5,6^

Previous examples use force-sensing instruments such as bipolar forceps^3^ or a blunt surgical dissector^7^ to measure forces applied by a surgeon in preclinical settings. Measurement of force or pressure is routinely performed using sensors which produce a reproducible and measurable change in parameters such as capacitance, current, voltage, or resistance when subject to a known mechanical load.^8^ The swift evolution of surgical robotics also yields force measurements^9^; however, robotics is not applicable to all types of surgery and might only be limited to certain parts of an operation. The previous examples of intraoperative force measurement are instrument and procedure specific and rarely used in routine clinical practice.

Translation of force quantification during surgery in real time requires further exploration and consideration of factors such as sterility, usability, and operative workflow. A novel approach is using sensorised surgical gloves. Sensorised gloves permit surgeons to use any instrument and perform any procedure or technique, while still achieving force measurement. Such sensorised gloves would be generalizable to any surgical specialty and likely straightforwardly adopted into operative workflow. We propose sensorised surgical gloves that can detect forces by incorporating a custom piezoresistive sensor, which responds to applied pressure by producing a change in the electrical resistivity of the material. Owing to the simple fabrication process, these sensors are durable and robust with high resolution to both static and dynamic pressure/strain.^8,10^

We aimed to (1) create a sensorised surgical glove to detect forces using a piezoresistive sensor, (2) determine forces at the sensorised glove-instrument and instrument-tissue interfaces, and (3) perform a preclinical surgical task with expert and novice surgeons.

## METHODS

### Creating a “Sensorised Surgical Glove” With Piezoresistive Force Sensors

**FIGURE 1.**
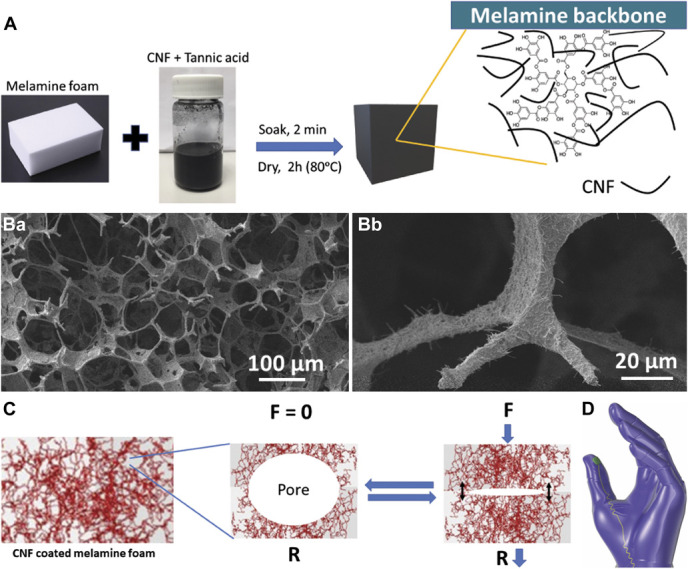
Summary of piezoresistive “sensorised surgical glove” fabrication. **A**, Schematic representation of the fabrication steps of the piezoresistive foam. **B**, Field emission scanning electron microscope of the piezoresistive foam: (a) the interconnected 3-dimensional pore structure of melamine with impregnated CNF and (b) magnified image showing fiber-like CNF adhered on the melamine framework. **C**, Mechanism of action of piezoresistive foam. Black arrows indicate CNF coming into contact with one another. **D**, Schematic of sensorised surgical glove. CNF, carbon nanofiber; F, force; R, resistance.

A custom-made conducting melamine foam-based soft piezoresistive sensor was fabricated and mounted on a surgical glove to measure forces during a surgical procedure (detailed fabrication process is presented in **Supplementary Material 1**, http://links.lww.com/NEU/D461). The piezoresistive sensor was mounted on the thumb of a surgical glove (Figure 1). Electrical resistance was measured using a digital multimeter (SDM3055), and initial force calibration was measured with a force plate (FP3, Biometric Ltd). Resistance readings were shown in real time in the laptop screen using LabVIEW (National Instruments). The resistance decreases when applying force and increases when releasing the force.

**FIGURE 2.**
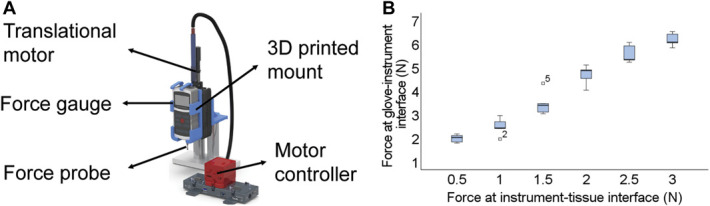
**A**, Laboratory experimental setup. **B**, Determining forces at the glove-instrument and the instrument-tissue interfaces from laboratory experiment. 3D, 3-dimensional.

A calibration setup converted the resistance measurements into force readings (Figure 2). The test setup and protocol used a motorized translation stage (PT1-Z8, Thorlabs) and a force gauge (M5-5 Mark-10) connected to a laptop. We increased the force applied in digitally controlled, high-resolution steps of 0.5N while recording resistance. After force calibration testing, force was plotted against resistance for each sensor used and a translational equation was fitted so that force data were provided in real time from measured change in resistance (**Supplementary Material 2**, http://links.lww.com/NEU/D461).

### Determining Forces at the Glove-Instrument and the Instrument-Tissue Interfaces

We investigated that the forces detected at the glove-instrument interface are discriminate of those applied at the instrument-tissue interface. We used the same laboratory setup (Figure 2) and measured the forces at the glove and at the tip of a surgical retractor, by pressing it against the force gauge's probe. The glove was first calibrated as previously described, and the difference in measured forces at both interfaces (glove-instrument and instrument-tissue) was then compared and plotted against each other. An equation was fitted to translate the force applied by the glove on the instrument to the force applied on the tissue by the tip of the instrument (**Supplementary Material 3**, http://links.lww.com/NEU/D461). The translational equation will vary from surgeon to surgeon depending on the lever arm's length and distance to the pivoting point.

### Microsurgical Task: “Stars the Limit”

A microsurgical task was used to illustrate function of the sensorised glove during neurosurgical procedures. Ethical approval was not required for this study because no patient or clinical data were collected, and this study was performed to plan and advise on future research.

### Participants

Six expert and 6 novice neurosurgeons were recruited from a university hospital. Surgeons were defined as novices if they had performed fewer than 5 surgical cases and experts if they had completed their surgical training.^7,11^ Owing to pragmatic constraints and lack of applicable pilot data, no power calculation was undertaken, but such a number was deemed appropriate based on previous similar studies.^7,11-13^

### Task

**FIGURE 3.**
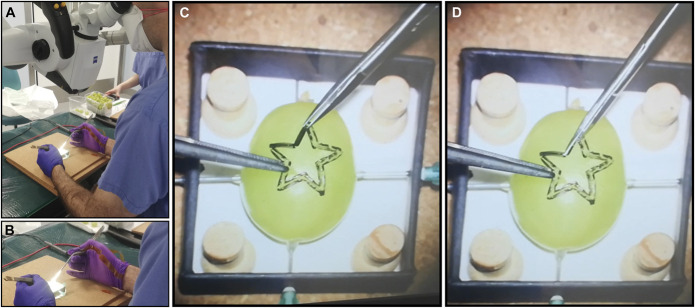
Photographs demonstrating experimental setup of **A**, expert performing grape dissection task with sensorised glove on the right hand. **B**, Magnified image of sensorised smart glove. **C**, and **D**, Demonstrate the validated dissection task “star's the limit” with dissection of the grape skin in the shape of a star, while leaving the grape flesh intact.

Participants performed a validated preclinical surgical task “Star's the limit.”^14,15^ A standardized star is drawn on a grape using a stencil with 5-mm edge length. Participants incise within the black line of the star and peel the star-shaped skin off the grape while minimizing damage to the grape flesh (Figure 3). Microscissors and forceps were provided to the participants. Each participant repeated the task 20 times. The OPMI PENTERO or KINEVO 900 (Carl Zeiss Co) operating microscopes were used. Surgeons were blinded to the real-time forces because this was a proof-of-concept study examining the feasibility of sensorised surgical gloves.

### Outcomes

We measured the median force applied (N), the median maximum force (N), the range of forces applied (N), and compared the differences between experts and novices. A “traffic light” system to compare the graphs obtained of force vs time for each test and surgeon. Forces ranging from 0 N to 4 N were “green,” 4 N to 8 N “yellow,” and any force above 8 N was “red.” These threshold values were chosen following our observations on the force applied by experts, to help visualize the spread of forces.

### Statistical Analysis

We used IBM SPSS v26.0. Outcome measures are presented as median ± IQR. Statistical analysis of differences was performed between forces used nonparametric tests (Mann-Whitney *U* test), and *P* < .05 was statistically significant.

## RESULTS

### Fabrication of the Sensorised Glove and Force Calibration

A piezoresistive sensor was integrated directly on surgical gloves (Figure 1). Force calibration was performed for each sensorised glove before use, and resistance vs force was plotted (Figure 2). We used translational equations for each sensor relating measured resistance and force applied (**Supplementary Material 2**, http://links.lww.com/NEU/D461).

### Determining Forces at the Glove-Instrument and the Instrument-Tissue Interfaces

We observed a difference in forces at the glove-instrument and instrument-tissue interfaces (Figure 2). For example, the measured force exerted at the instrument-tissue interface of 3.00 N corresponded to a measured force of 8.37 N (6.47–8.41 N) at the glove-instrument interface. This is shown and calculated as an example for a particular surgeon who repeated the experiment to determine the relationship of the forces at both interfaces 10 times. Nevertheless, as stated above, this relationship will vary depending on the point at which the instrument is held and the way in which it is held. Calibrating for each surgeon and the technique used when holding the instrument may lead to accurate relationships between the force applied on the glove and the force applied on the tissue for each user.

### “Stars the Limit” Microsurgical Task Performed With the Sensorised Glove

**FIGURE 4.**
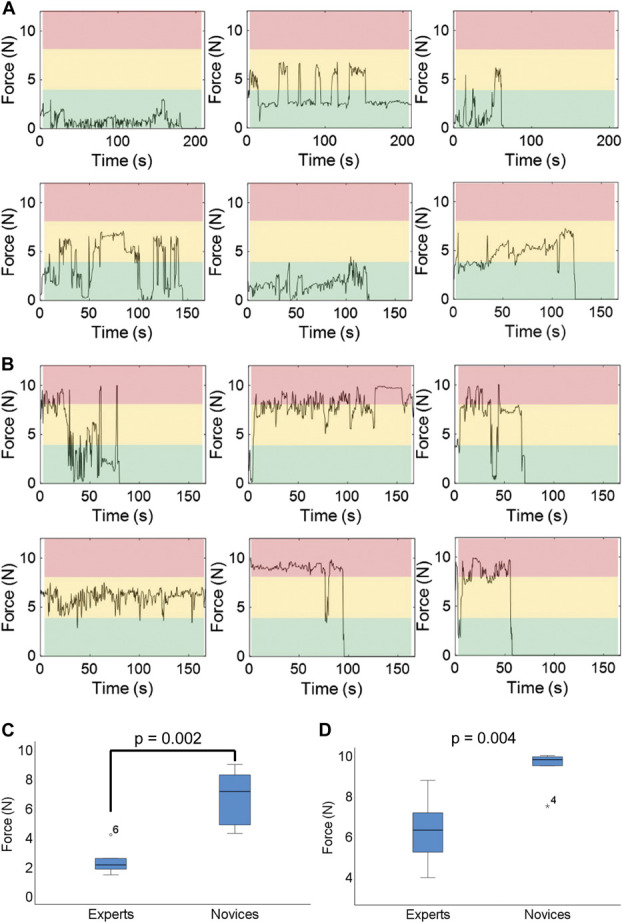
Summary of expert **A**, and novice **B**, exerted forces during grape dissection task. **C**, Median force applied by experts and novices (*P* = .002). **D**, Median maximum force applied by experts and novices (*P* = .004).

Expert surgeons (n = 6; M:F 4:2) had a median 10.0 years of surgical experience (IQR: 8.9-24.0 years). Novice surgeons (n = 6; M:F 3:3) had a median 0.3 years of surgical experience (IQR: 0.2-2.1 years). Expert and novice surgeons completed the microsurgical grape dissection task while wearing the sensorised glove. There was a significant difference (*P* = .002) between the median force applied by experts and novices. The median force applied by experts was 2.14 N (1.76-2.98 N) and novices was 7.15 N (4.72-8.45 N). There was also a significant difference (*P* = .004) in the maximum forces applied by experts and novices (Figure 4; **Supplementary Material 4**, http://links.lww.com/NEU/D461; **Supplementary Figures 2-4**, http://links.lww.com/NEU/D461); Video 1; Video 2. The average maximum force applied by experts was 6.32 N (4.92-9.00 N) and novices was 9.80 N (7.57-9.95 N) (Table; Figure 4).

**TABLE.** Summary of Measured Force Data by the Smart Glove from the Surgical Grape Dissection Task “Star's the Limit”.

	Median force (IQR) (N)	Median maximum force (IQR) (N)
Expert 1	1.46 (0.61-1.65)	3.97 (3.58-4.58)
Expert 2	2.58 (2.33-2.99)	7.17 (6.86-7.41)
Expert 3	1.99 (1.02-2.31)	5.74 (3.93-6.59)
Expert 4	2.29 (1.11-4.13)	6.89 (6.29-7.48)
Expert 5	1.85 (1.60-2.40)	5.23 (4.45-6.74)
Expert 6	4.20 (4.09-4.48)	8.77 (8.26-8.89)
	2.14 (1.76-2.98)	6.32 (4.92-9.00)
Novice 1	4.29 (2.28-4.58)	10.00 (9.86-10.05)
Novice 2	7.36 (6.39-8.32)	9.94 (9.39-10.48)
Novice 3	6.94 (6.30-7.58)	9.50 (9.21-9.79)
Novice 4	4.87 (4.63-5.63)	7.50 (7.42-7.57)
Novice 5	8.98 (8.41-9.54)	9.74 (9.54-9.86)
Novice 6	8.27 (7.90-9.13)	9.86 (9.45-9.91)
	7.15 (4.72-8.45)	9.80 (7.57-9.95)

N, Newton.

## DISCUSSION

### Principal Findings

To the best of our knowledge, this is the first study to create a sensorised surgical glove capable of measuring forces during surgery. We developed a piezoresistive sensor that can be mounted on surgical gloves to determine force applied during surgical interventions in real time. The sensorised glove was calibrated across a range of forces (0-10 N) and demonstrated a predictable difference in measured force between the glove-instrument and instrument-tissue interface. Importantly, the sensorised glove detected a significant difference between the median and maximum force exerted by expert and novice surgeons during a validate surgical task (Figure 4).

### Comparison With the Literature

Previous literature demonstrates that surgical proficiency correlates with clinical outcomes^16^ and that instrument-tissue forces can act as a measure of surgical performance.^3,4,17,18^ Quantitative metrics for surgical performance such as morbidity or mortality are surrogate measures and subject to bias by confounding parameters not necessarily related to surgical skill.^19^ Furthermore, surgical simulation demonstrates that >50% of errors by surgical trainees are attributable to excessive force.^20^ Previous instruments to detect intraoperative forces include bipolar forceps^3^ or a blunt surgical dissector.^7^ Numerous robotic force measurement devices exist, but they are large, highly complex, and costly.^21^ Our sensorised glove provides an innovative solution to measure intraoperative forces that can be adapted pan-specialty and applied to all surgical procedures. This will create a platform to measure forces during operations and will provide quantitative feedback for surgeons and trainees.

Consideration should be given to different intraoperative forces between surgical procedures because these are known to vary significantly. For example, in a recent review, the forces exerted in ophthalmological procedures were found to be lowest (0.04 N), whereas orthopedics the highest (210 N).^2^ Within a particular procedure, forces exerted may similarly vary significantly according to the surgical step, depending on factors such as the instrument used, the surgical action performed, and the tissue being manipulated. In each case, the optimal is a careful balance between surgical effectiveness and safety. Further research could examine the range of forces to further our understanding about “safe” intraoperative forces at each operative step. This could be achieved by deconstructing operations into operative workflows, exemplified by pituitary adenoma^22^ and vestibular schwannoma resection,^23^ and creating a “safe force range” in real time. This might improve surgical safety because awareness of force exerted by the operating surgeon in real-time has been shown to decrease intraoperative forces^24,25^ while not significantly disturbing the workflow of the procedure.^21^

The sensorised glove could eventually connect to an audio feedback system that would alert the surgeon of excessive force or allow for set upper force limits to improve surgical safety and thus improve patient safety during operations.^5,6^ A future research direction could investigate the human factors attributable to surgery, such as fatigue, stress, and cognitive overload, and how this might affect the intraoperative forces exerted. This could begin with unblinding the participant to the forces to establish whether the real-time feedback improved learning or performance.

Sensorised gloves continue to suffer from technical issues, and no single sensor has been integrated into routine practice. Burdea et al,^26^ measured grasping force using a tactile sensing glove. However, the ultrasonic force sensors used produced excess noise because of environmental interference.^26^ Similarly, a resistor-based sensor was also developed to measure grasping force, but their performance is hindered by the electromagnetic interference from the wearer.^27^ Separately, Nikonovas et al,^28^ developed a conductive ink-based sensor mounted glove to measure force, but they are slow to respond and hence unsuitable for dynamic measurements. Piezoresistive fabric-based sensor gloves, such as the 1 used in our sensorised surgical glove, provide novel utility as it conforms to the human hand, although do exhibit complex hysteresis and drift behavior which decreases sensitivity.^29^ Other parameters such as use of low-cost materials, robustness, scalability, repeatability, and sterilization need to be considered for specific surgical interventions. Our sensorised surgical glove exhibits the essential attributes, and its ability to determine intraoperative forces serves as a new, simple and sustainable way to modulate and control instrument-tissue interactions toward safe outcomes. The sensorised glove is scalable, and the piezoresistive foam is fabricated using low-cost and biocompatible materials such as melamine, carbon nanofiber, and tannic acid. The foam structure is porous to provide sufficient robustness, with the soft nature of the foam enables conformability with a gloved human hand or any medical tools, and multiple sensors can be used—resulting in a generalizable sensorised surgical glove. Furthermore, all the sensor components have decomposition temperatures >200 °C^30,31^ which can tolerate routine sterilization—a prerequisite for medical tools.

### Strengths and Limitations

The strengths of this study include the strong multidisciplinary approach and collaboration between neurosurgeons and nanoengineers. This yielded iterative improvement in technical aspects and provided a simulated realistic environment to answer and explore appropriate clinical questions. The robust methodology from development of the glove, laboratory experiment, and preclinical grape dissection task provides a logical innovation process. Furthermore, the simulated task is surgically relevant, previously validated, and provides useful force data that can be incorporated into future studies.

A limitation is related to the piezoresistive sensor. Because this is a newly developed device, the sensor might not be optimally placed to measure all forces for all instruments. Furthermore, our sensor was located at a single point on the surgical glove (Figure 1D). However, over time and through iterative improvement, the sensor placement will be optimized. For example, a body-mounted sensor could be constructed to reduce the need to be connected to a fixed device by wire. In addition, it is unclear whether the profile of the sensors can impair the haptic feedback for the operating surgeon, which could interfere with a real-world operation. Although the grape dissection task was a useful starting point, it is low fidelity and does not simulate complications, which, in real life, might lead to increased force exertion as the operating surgeon is under pressure. Therefore, further work could use the smart gloves in high-fidelity simulation models to increase the validity of the findings.

### Next Steps

This relates to sensor design, task, and translation. We will modify the sensor to make it smaller and thinner. Efficient packaging of electrical interconnects will improve glove manoeuvrability. This proof-of-concept study holds strong potential that needs further development using higher fidelity models with more neurosurgical-focused tasks. Translation into sensor-embedded “traditional” surgical gloves and performing surgical tasks on patients is planned as future steps to aid in broader neurosurgical interventions.

## CONCLUSION

We developed a sensorised surgical glove to detect forces across 0 to 10 N. The sensorised glove detected a significant difference between experts and novices during a surgical task. The sensorised glove provides a mechanism to produce real-time data to optimize intraoperative forces and improve patient safety. Force analysis can be used as an adjunct to surgical training and assessment. The sensorised glove has potential applications within neurosurgery and other surgical specialties because it can integrate into existing operative workflow.

## Notes

**Supplementary Material 1.** Fabrication of custom-made piezoresistive sensors. **Supplementary Material 2.** Calibration of sensors. **Equation 1.** Translational power function obtained through calibration relating measured resistance from the sensors to the force applied on the glove. **Supplementary Material Figure 1.** Plot obtained after the calibration test of a given sensor showing force applied against resistance change measured and power function fit. **Equation 2.** Translational power function obtained through calibration relating measured resistance from the sensors to the force applied on the glove. **Supplementary Material 3.** Relating force on the glove-instrument interface to the force applied on the instrument-tissue interface. **Equation 3.** Translational linear equation relating force on the glove-instrument interface to the force applied on the instrument-tissue interface. **Supplementary Material 4.** Maximum force application with the sensorised surgical glove. **Supplementary Material Figure 2.** Figure showing the expert's hand a different tilted positions: (a) nontilted position, (b) very slightly tilted intermediate position, and (c) tilted position reached. **Supplementary Material Figure 3.** Figure showing higher forces applied by experts 1, 3, 4, 5, and 6 on the most tilted position (red circle) and by experts 3, 4, 5, and 6 during dissection (blue rectangle). **Supplementary Material Figure 4.** Dissection of the grape's skin: (a) expert dissecting the grape's skin after having cut through the star's edges, (b) image shown on the microscope screen while the expert is dissecting the grape, and (c) expert dissecting the grape's skin after having cut through the star's edges.
